# The viral agents of Acute Encephalitis Sydrome in Ben Tre province 2008-2010

**Published:** 2011-01-10

**Authors:** Tran Ngoc Huu, Luong Chan Quang, Do Kien Quoc, Phan Van Tinh, Huynh Thi Kim Loan, Nguyen Thi Thanh Thao, Phan Van Tu, Vu Thi Que Huong

**Affiliations:** 1 Pasteur Institute of Ho Chi Minh City, Vietnam

## 

Since 2005, Arboviruses and Enteroviruses have been confirmed as some of pathogens causing acute meningo-encephalitis in children in the South of Vietnam. Therefore, they have been selected in priority into a sentinel surveillance and investigation system of emerging infectious diseases in Ben Tre province during 2008-2010. The surveillance system for viral Acute Encephalitis Syndrome (AES) was established in 02 sentinel hospitals in Ben Tre province, Vietnam. All AES cases with macroscopic appearance corresponding to viral AES were enrolled into the surveillance. Their specimens (CSF, paired sera and feces) were only collected after patients or their legal guarantees had signed in the written ICF. The second serum of paired sera was collected on the field in case patient had discharged. MAC-ELISA was applied for detecting Arboviruses (such as Japanese encephalitis virus and Dengue viruses), and RT-PCR was used for detecting Enteroviruses, especially Enterovirus type 71.

From August 2008 to June 2010, 138 AES cases were enrolled into the surveillance system with 138 CSF, 276 sera and 138 feces specimens. Out of 138 reported cases from the surveillance system, 60 (43.48%) AES cases had positive testing results with viral agents, including: 7 (5.07%) Japanese encephalitis virus, 6 (4.35%) Dengue viruses, and 47 (34.06%) Enteroviruses. No case of infecting Enterovirus type 71 was detected. Epidemiological analysis shown that Enteroviruses circulated all year round, Dengue viruses circulated from August to October in accordance to the Dengue epidemic season in the South of Vietnam and Japanese encephalitis virus circulated sporadically in a year. Moreover, distribution of viral agents specific by age groups shown that age group of 0 – 14 (11.76%) and over 60 (18.18%) were the most susceptible for infecting Japanese encephalitis virus.

The surveillance system has provided valuable information as a baseline for future studies on viral agents of AES and for implementing Japanese encephalitis vaccination campaigns in Ben Tre province. The implementation in reality also raised the needs of developing and applying diagnostic tests with high sensitivity and specificity such as NS1 antigen detection for Japanese encephalitis virus and multiplex RT-PCR for Enteroviruses.

